# A comparison among optimization software to solve bi-objective sectorization problem

**DOI:** 10.1016/j.heliyon.2023.e18602

**Published:** 2023-07-29

**Authors:** Aydin Teymourifar

**Affiliations:** Universidade Católica Portuguesa, Católica Porto Business School, Centro de Estudos em Gestão e Economia Porto, Portugal

**Keywords:** Multi-objective optimization, Sectorization, Mixed integer non-linear programming, GAMS, CPLEX, Lingo, Python, Pulp, Pymoo, GA, NSGA-II

## Abstract

In this study, we compare the performance of optimization software to solve the bi-objective sectorization problem. The used solution method is based on an approach that has not been used before in the literature on sectorization, in which, the bi-objective model is transformed into single-objective ones, whose results are regarded as ideal points for the objective functions in the bi-objective model. Anti-ideal points are also searched similarly. Then, using the ideal and anti-ideal points, the bi-objective model is redefined as a single-objective one and solved. The difficulties of solving the models, which are basically non-linear, are discussed. Furthermore, the models are linearized, in which case how the number of variables and constraints changes is discussed. Mathematical models are implemented in Python's Pulp library, Lingo, IBM ILOG CPLEX Optimization Studio, and GAMS software, and the obtained results are presented. Furthermore, metaheuristics available in Python's Pymoo library are utilized to solve the models' single- and bi-objective versions. In the experimental results section, benchmarks of different sizes are derived for the problem, and the results are presented. It is observed that the solvers do not perform satisfactorily in solving models; of all of them, GAMS achieves the best results. The utilized metaheuristics from the Pymoo library gain feasible results in reasonable times. In the conclusion section, suggestions are given for solving similar problems. Furthermore, this article summarizes the managerial applications of the sectorization problems.

## 1 Introduction

Almost all optimization software and solvers have evolved considerably over time, but they still have significant differences [1]. Therefore, in the literature, there are studies comparing solvers [2], [3], [4], [5], [6], [7]. Likewise, some studies compare the evolutionary algorithm (EA) with solvers and discuss the advantages and disadvantages [8], [9], [10]. In this study, a comparison is made between optimization software to solve a sectorization problem (SP). The goal of SPs is to split a large region into smaller ones for administrative purposes [11], [12], [13], [14]. However the main contribution of the study to the literature is the comparison among optimization software, it has other novelties, which can be summarized as follows:

• A new bi-objective (BO) model is defined for SP, which is nonlinear (NL).
• A new approach is applied to solve the model, in which, at first, the BO model is divided into two single-objective (SO) ones, which are also NL.
• The goal of the division is to gain ideal points (IDPs) and anti-ideal points (ADPs) for the objective functions in the BO model.
• The difficulties and complexities of solving the model such as the nonlinearity of objective functions and of some constraints are discussed.
• Moreover, models are linearized for possible easier solutions.
• The outputs of the linearized models are considered as IDPs and ADPs of the objective functions in the BO model.
• The results of the linear (L) and NL models are supposed to be the same if they are optimal. However, this is also a way to evaluate the computational performance of the solvers.
• The BO model is re-defined based on the LP-metric method, using IDPs and ADPs for the objective functions.

Though the employed solution approach is well-known in optimization topics, it is employed for the first time in the sectorization literature.

As a contribution to the literature, a comparison is made between different software for solving MO SP. For mathematical models, different solvers like Python's Pulp library, Lingo, IBM ILOG CPLEX Optimization Studio, and GAMS are used. There is a similar study in the literature comparing optimization tools for solving SPs [15]. But it doesn't report the results of GAMS, IBM ILOG CPLEX Optimization Studio, and Lingo. Furthermore, the solution approach in this study is different. From SO metaheuristics available in the Pymoo library of Python, a Genetic Algorithm (GA), and from MO ones the Non-dominated Sorting Algorithm (NSGA-II) are employed [16].

**Figure 1:**
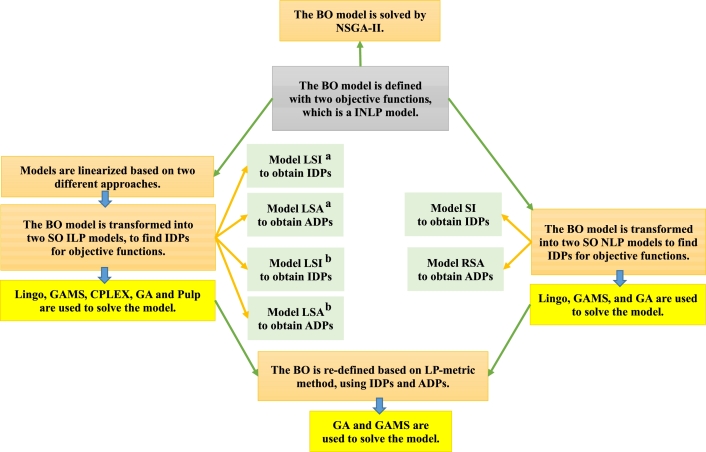
A summary of applied methods to the BO model.

A summary of the methods that are applied in this paper is presented in Fig. 1, whose details are described in Section 4. The used abbreviations can be found in the appendix.

The other parts of the paper are organized as follows: Section 2 summarizes the related literature. The problem definition and solution method are respectively described in Sections 3 and 4. In the experimental results section, based on generated benchmarks, the operated software and algorithms are compared. Conclusion and future works form the last part of the article, where comments and suggestions for solving similar models are offered as well as several research directions.

## 2 Literature review

Koch et al. [1] report that from 2001 to 2020 linear programming (LP) and mixed integer linear programming (MILP) solvers became about 180 and 1000 times faster, respectively. Furthermore, benchmarks that couldn't be solved years ago are now solved in seconds [1]. Despite all these advancements, occasionally unexpected variability affects the performance of mixed integer programming (MIP) solvers. This issue has been observed for decades. Lodi and Tramontani [2] discuss the causes of this phenomenon. Therefore, the performance of solvers for various problems is of interest to researchers. Neumaier et al. [3] compare the performance of constrained optimization solvers over 1000 benchmarks from literature, which contain 1000 variables. Kronqvist et al. [4] make a review and comparison between mixed integer non-linear programming (MINLP) solvers based on a set of 335 benchmarks, which are divided into subsets based on the continuous relaxation gap, the degree of nonlinearity, and also the relative number of discrete. The acquired results provide information about which solver or method is suitable for which type of benchmarks. Meindl and Templ [5] compare various solvers on LP benchmarks. Luppold et al. [6] investigate the effects of different types of variables, constraints, and logical expressions on the performance of solvers. Describing the complexity of hard real-time systems with large numbers of constraints, Guasque and Balbastre [7] emphasize the importance of heuristic methods to obtain feasible solutions for them. They also make a comparison between CPLEX and Gurobi.

In this study, the comparison between optimization software is done over SPs. As stated in the previous paragraph, the type of problem and the solution method affect the performance of employed solvers. Therefore, this section summarizes the literature on different types of SPs and the utilized methods for their solution. The main objective of SPs is to divide a vast district into smaller and balanced areas. SPs are mostly formulated as multi-objective (MO) models and they have applications in numerous fields. Some of the problems modelled based on sectorization, the used solution approaches for them, and whether the model is MO or not appear in Table 1. The used abbreviations can be found in the appendix.

**Table 1:** Some of the problems modeled based on sectorization and employed approaches to solve them.

Problem	Solution Approach	MO model
Political districting [11]	ILP	
Facility location [12]	GP	✓
Location allocation [13]	Metaheuristics	
Location routing [17]	Heuristic and metaheuristic	
Location routing [18]	Heuristic	
Supply chain network [19]	Metaheuristic	
Elderly care service districting [20]	MINLP	✓
Emergency medical service districting [21]	Metaheuristic	
Power distribution networks [22]	Metaheuristic	✓
Police districting [23]	Literature review	
Commercial districting [24]	Metaheuristic	
School districting [25]	Heuristic	
Air traffic control [26]	Heuristic and metaheuristic	
Air traffic control [27]	Human factors issues	
Air traffic control [28]	Constraint programming	
Air traffic control [29]	Metaheuristic	✓
Air traffic control [30]	Simulation	
Air traffic control [31]	Metaheuristic	✓
Telecommunications [32]	Simulation	
Telecommunications [33]	IQP, MIP	
Water distribution networks [34]	MINLP and metaheuristic	✓

SPs have managerial implications, some of which are shown in Table 2.

**Table 2:** Managerial applications of SPs.

Application	Filed
Decreasing wastes [35]	Water management
Managing capacity [36]	Air traffic management
Improving utility [37]	Healthcare management

**Figure 2:**
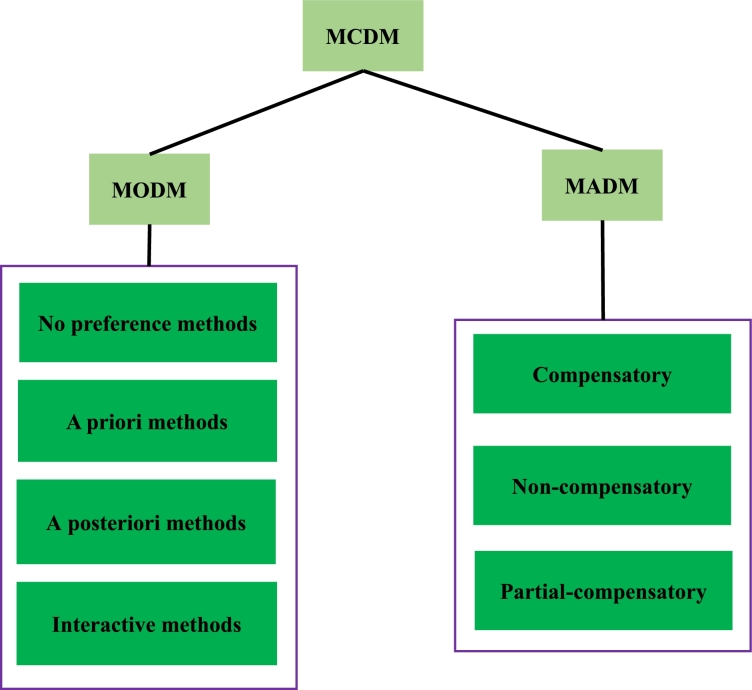
Classification of MCDMs [38], [39], [40], [41].

MO models fall into multi-criteria decision-making (MCDM) techniques. As seen in Fig. 2, MCDM techniques can be categorized as multi-attribute decision-making (MADM) and multi-objective decision-making (MODM) subcategories that respectively have discrete and continuous decision spaces. MODMs are divided into four categories based on the role of the decision maker (DM). In the first class, which is a neutral approach, there is no preference information from the decision-maker. The LP-metric method is in this category, which trade-offs between desired and undesired solutions and in this way converts an MO model to an SO one. In other classes, preference information is received from the DM before, after, or interactively during the resolution process. MADM techniques can be divided into three categories: compensatory, non-compensatory, and partial compensatory. In compensatory approaches, some negative attributes can be compensated by positive ones [38], [39], [40], [41].

In the literature, the LP-metric or the methods designed based on IDPs and ADPs have not been used to solve SPs, however, they have been applied for the solution of several problems, some of which are summarized in Table 3 [42].

**Table 3:** Problems solved based on IDPs and ADPs in the literature.

Problem	Combined Method with IDPs and ADPs
Location selection [43]	Fuzzy decision making
Efficiency evaluation [44]	Data envelopment analysis
Supplier selection [45], [46]	MCDM
Wind site selection [47]	Fuzzy MCDM
Portfolio asset management [48]	MODM

Some of the problems in the literature solved based on other techniques of MCDM are summarized in Table 4.

**Table 4:** Some of the problems solved based on MCDM techniques.

Problem	Method	Category of the Method
Air traffic control [49]	Weighted sum	MODM, a priori methods
Water distribution network [50]	Lexicographic	MODM, a priori methods
Air traffic control [51]	Scalarization	MODM, a priori methods
Water distribution network [52]	AHP	MADM, compensatory

There are also other MCDM techniques in the literature that have not been used for SPs. For example, from MODM, a priori methods: lexicographic max-min [53] *ϵ*-constraint, goal programming [54], surrogate worth trade-off method (SWTM) [55], from interactive methods [56], step method (STEM) [57], from MADM, compensatory methods: the technique for order preference by similarity to ideal solution (TOPSIS), analytical network process (ANP) [58], [59], [60], [61] and from MADM, non-compensatory methods: Max-Max, Min-Max [62] have not been employed for SPs solution.

## 3 Problem definition

In the problem, it is assumed that there are clients, to be assigned to some service centers, whose numbers and coordinates are predetermined. Each service center and its assigned clients form a sector. Each client is assigned to only one sector. Each of the formed sectors has at least one client. The number, coordinates, and service centers are known beforehand. Each client has a predefined demand.

Some of the used notations are summarized in Table 5.

**Table 5:** Used notations.

Notation	Description
*n*	Total number of clients
*k*	Total number of sectors (and service centers)
*Q*	Target number of clients per sector
*m*	Number of objective function
*Z*_*ij*_	Decision variable about assignment of client *i* to sector *j*
*d*_*ij*_	Euclidean distance between client *i* and the service center of sector *j*
*Dc*_*j*_	Total distance of clients of sector *j* from the related service center
$\overset{¯}{Dc}$	Average of the distance of clients from the service center of sectors
*de*_*i*_	Demand of client *i*
*De*_*j*_	Total demand of clients in sector *j*
$\overset{¯}{De}$	Average of the demand of clients in sectors
*τ*_*eqds*_	Parameter for the equilibrium of distances in sectors
*τ*_*eqde*_	Parameter for the equilibrium of demand in sectors
*f*_1_	Objective function related to compactness
*f*_2_	Objective function related to equilibrium of demands in sectors
*l*_*m*_	Linearized form of *f*_*m*_
$f_{m}^{⁎}$	Ideal point for *f*_*m*_
$f_{m}^{⁎⁎}$	Anti-ideal point for *f*_*m*_
$l_{m}^{⁎}$	Ideal point for the linearized form of *f*_*m*_
$l_{m}^{⁎⁎}$	Anti-ideal point for the linearized form of *f*_*m*_
*f*	Bi-objective function related to compactness and equilibrium
*V*	Positive variables for linearizing the objective functions

The binary decision variable of the model is as in Eq. (1).

(1) $$Z_{ij}=\left\{ \begin{array}{cc} 1, & \text{if }\;client\;i\;is\;assigned\;to\;sector\;j\\ 0, & \text{otherwise}. \end{array}\right.$$

 i=1,...,n, j=1,...,k.

### 3.1 BO model

To be minimized, the objective function related to the equilibrium of distances in sectors is defined as in Eq. (2).

(2) $$f_{1}=\sum_{j=1}^{k}|Dc_{j}-\overset{¯}{Dc}|$$

where $Dc_{j}=\sum_{i=1}^{n}d_{ij}\times Z_{ij}$, $\overset{¯}{Dc}=\frac{\sum_{j=1}^{k}Dc_{j}}{k}$.

To be minimized, the objective function related to equilibrium of demands in sectors is defined as in Eq. (3).

(3) $$f_{2}=\sum_{j=1}^{k}|De_{j}-\overset{¯}{De}|$$

where $De_{j}=\sum_{i=1}^{n}de_{i}\times Z_{ij}$, $\overset{¯}{De}=\frac{\sum_{j=1}^{k}De_{j}}{k}$.

The purpose of $f_{1}$ and $f_{2}$ is to create more balanced sectors in terms of distance and demand.

In this way, to be minimized, the BO function of the model is defined as in Eq. (4).

(4) $$f\;=(f_{1},f_{2})$$

Each client is assigned to just one sector, which is satisfied by Constraint (5).

(5) $$\sum_{j=1}^{k}Z_{ij}=1,\;i=1,...,n$$

As specified in Constraint (6), at least one client is assigned per sector.

(6) $$\sum_{i=1}^{n}Z_{ij}\geq 1,\;j=1,...,k$$

Furthermore, as defined in Eq. (1), *Z* is a binary variable, which is considered as a constraint in the model.

It is expected that the equilibrium is yielded in terms of distances and demands between the formed sectors, which is provided by Constraints (7)-(8).

(7) $$|Dc_{j}-\overset{¯}{Dc}|\leq \overset{¯}{Dc}(1-\tau_{eqds}),\;j=1,...,k$$

(8) $$|De_{j}-\overset{¯}{De}|\leq \overset{¯}{De}(1-\tau_{eqde}),\;j=1,...,k$$

$\tau_{eqds}$, and $\tau_{eqde}$ are parameters for the equilibrium of distances and demand in sectors. The reason for using Constraints (7)-(8) can be explained as follows: The total deviations of $Dc_{j}$ and $De_{j}$, j=1,...,k, from their averages are minimized by applying the objective functions $f_{1}$, and $f_{2}$. Whereas using Constraints (7)-(8), more desired solutions are provided by defining upper limits to the deviation value for each sector. If the objective functions were defined based on variance or standard deviation instead of absolute value, they would have the same influence, but upper limits of deviations could not be guaranteed.

The complete form of the basic NL model can be expressed with Statements (9)-(15).

(9) $$min\sum_{j=1}^{k}|\sum_{i=1}^{n}(d_{ij}Z_{ij}-\frac{1}{k}\sum_{p=1}^{k}d_{ip}Z_{ip})|$$

subject to

(10) $$min\sum_{j=1}^{k}|\sum_{i=1}^{n}d_{ij}(Z_{ij}-\frac{1}{k}\sum_{p=1}^{k}Z_{ip})|$$

(11) $$\sum_{j=1}^{k}Z_{ij}=1,\;i=1,...,n$$

(12) $$\sum_{i=1}^{n}Z_{ij}\geq 1,\;j=1,...,k$$

(13) $$|\sum_{i=1}^{n}(d_{ij}Z_{ij}-\frac{1}{k}\sum_{p=1}^{k}d_{ip}Z_{ip})|\leq \frac{1}{k}(1-\tau_{eqds})\sum_{i=1}^{n}\sum_{p=1}^{k}d_{ip}Z_{ip},\;j=1,...,k$$

(14) $$|\sum_{i=1}^{n}d_{ij}(Z_{ij}-\frac{1}{k}\sum_{p=1}^{k}Z_{ip})|\leq \frac{1}{k}(1-\tau_{eqde})\sum_{i=1}^{n}\sum_{p=1}^{k}Z_{ip},\;j=1,...,k$$

(15) $$Z_{ij}\in \left\{0,1\right\},\;i=1,...,n,j=1,...,k$$

## 4 Solution method

Before describing the solution method, some concepts are explained. Vectors of nadir [63] and ideal objectives are used in the literature on MO optimization [64], which are not the same as IDPs and ADPs in this work. The next definitions need to be made to clarify the differences:

Definition 4.1 In a minimization problem the following conditions must be valid for the solution $z^{1}$ to dominate $z^{2}$:

∀m∈{1,2} $f_{m}(z^{1})\leq f_{m}(z^{2})$ & ∃m∈{1,2}  $f_{m}(z^{1})<f_{m}(z^{2})$.

Definition 4.2 $z^{Pa}$ is a Pareto optimal or an efficient solution if it is not dominated by any other solution.

In the field of MO optimization, two distinct terms, “weak efficiency” and “strict efficiency,” are distinguished. A solution is considered weakly efficient if it is not dominated by another one in the solution space. This means that, given a certain objective, a weakly efficient solution may be dominated by another one. Yet, a solution is considered strictly efficient if it is not dominated by any other solution according to any objective. In this research, weakly efficient solutions are referred to as efficient solutions.

Definition 4.3 The set of efficient solutions forms the Pareto front, which is denoted as $Z^{Pa}$.

Definition 4.4 The Pareto front is bounded by the vector of nadir and ideal objectives, which are defined as $Sup_{z^{Pa}\in Z^{Pa}}f_{m}(z^{Pa})$, and $Inf_{z^{Pa}\in Z^{Pa}}f_{m}(z^{Pa})$, ∀m∈{1,2}, respectively.

The IDPs and ADPs in this study are the outputs obtained from SO models, which are operated in the LP-metric method to transform the MO model into an SO one.

It can be stated that the LP-metric method sets weights for objectives and hence is similar to the weighted sum method. The weighted sum approach is in the category of a priori methods in Fig. 2, because weights are usually defined by the DM before the solution process. Whilst in the LP-metric method, the weights are determined during the solution procedure.

According to Ref. [38], optimal solutions of the weighted sum problem with positive (non-negative) weights are always (weakly) efficient. Furthermore, all (weakly) efficient solutions are optimal solutions of scalarized problems with positive (non-negative) weights under convexity assumptions.

It should be noted that integer programming models are considered non-convex, thus convexity assumptions are not valid for them.

In the next subsection, the solution method is described, which has not been used in previous studies about SP. In this method, at first, the BO model is converted into two SO models, from which IDPs and ADPs are found for the objective functions. Then the BO model is redefined and transformed to an SO model by using IDPs and ADPs. L versions of the models are also utilized.

### 4.1 Dividing the BO model into two NL SO models

The goal of the division is to determine IDPs and ADPs for objective functions.

#### 4.1.1 $SI_{1}$, SO model to find an IDP for $f_{1}$ in the BO model

To be minimized, the objective function of this model is $f_{1}$, as defined in Eq. (2). The optimal solution of this model, $f_{1}^{⁎}$, is an IDP for $f_{1}$ in the BO model.

#### 4.1.2 $SI_{2}$, SO model to find an IDP for $f_{2}$ in the BO model

To be minimized, the objective function of this model is $f_{2}$, as defined in Eq. (3). The optimal solution of this model, $f_{2}^{⁎}$, is an IDP for $f_{2}$ in the BO model.

Constraints (5)-(8) are valid in the $SI_{1}$ and $SI_{2}$ models.

#### 4.1.3 $SA_{1}$, SO model to find an ADP for $f_{1}$ in the BO model

When the objective function, $f_{1}$, is to be maximized instead of be minimized, the optimal solution, $f_{1}^{⁎⁎}$, is an ADP for $f_{1}$ in the BO model.

#### 4.1.4 $SA_{2}$, SO model to find an ADP for $f_{2}$ in the BO model

To be maximized, the objective function of this model is $f_{2}$, defined as in Eq. (3). The optimal solution of this model, $f_{2}^{⁎⁎}$, is an ADP for $f_{2}$ in the BO model.

Models $SA_{1}$ and $SA_{2}$ comprise Constraints (5)-(6). However, they don't contain Constraints (7)-(8), since they define an upper limit, which is not meaningful for maximization.

### 4.2 Linearizing SO models

In this subsection, the SO models are linearized according to two different approaches. The number of variables in these two approaches is different. They are used to demonstrate the influence of the number of variables.

#### 4.2.1 $LSI_{1}^{a}$, linearized SO model based on the first approach to find an IDP for $f_{1}$ in the BO model

Using non-negative variable $V_{1_{j}}$ objective function $f_{1}$ is linearized.

(16) $$l_{1}=\sum_{j=1}^{k}V_{1_{j}}$$

(17) $$Dc_{j}-\overset{¯}{Dc}\leq V_{1_{j}},\;j=1,...,k$$

(18) $$\overset{¯}{Dc}-Dc_{j}\leq V_{1_{j}},\;j=1,...,k$$

(19) $$V_{1_{j}}\;\geq 0,\;j=1,...,k$$

(20) $$Dc_{j}-\overset{¯}{Dc}\leq \overset{¯}{Dc}(1-\tau_{eqds}),\;j=1,...,k$$

(21) $$\overset{¯}{Dc}-Dc_{j}\leq \overset{¯}{Dc}(1-\tau_{eqds}),\;j=1,...,k$$

(22) $$De_{j}-\overset{¯}{De}\leq \overset{¯}{De}(1-\tau_{eqde}),\;j=1,...,k$$

(23) $$\overset{¯}{De}-De_{j}\leq \overset{¯}{De}(1-\tau_{eqde}),\;j=1,...,k$$

Constraints (20)-(23) are the linear versions of Constraints (7)-(8). Constraints (5)-(6) are also included in this model. The optimal solution of this model, indicated as $l_{1}^{⁎}$, is an IDP for $f_{1}$ in the BO model.

#### 4.2.2 $LSI_{1}^{b}$, linearized SO model with the second approach to find an IDP for $f_{1}$ in the BO model

Using non-negative variables $V_{1_{j}}$ and $V_{2_{j}}$, j=1,...,k, objective function $f_{1}$ is linearized.

(24) $$l_{1}=\sum_{j=1}^{k}V_{1_{j}}+\sum_{j=1}^{k}V_{2_{j}}$$

(25) $$Dc_{j}-\overset{¯}{Dc}-V_{1_{j}}+V_{2_{j}}=0,\;j=1,...,k$$

(26) $$V_{1_{j}}\;and\;V_{2_{j}}\geq 0,\;j=1,...,k$$

To be minimized, the objective function of this model is determined as in Eq. (24). The optimal solution of this model, denoted as $l_{1}^{⁎}$, is an IDP for $f_{1}$ in the BO model. Constraints (5)-(6) and (20)-(23) are also contained in this model, as well as Constraints (25)-(26).

In the $LSI_{1}^{a}$ model, there are additionally 2*k* variables due to $V_{1_{j}}$ and $V_{2_{j}}$, while there are further *k* variables in the $LSI_{1}^{b}$ model because of $V_{1_{j}}$ or $V_{2_{j}}$. Moreover, in both models, 7*k* constraints are added.

#### 4.2.3 $LSI_{2}^{a}$, linearized SO model based on the first approach to find an IDP for $f_{2}$ in the BO model

Using non-negative variable $V_{2_{j}}$ objective function $f_{2}$ is linearized.

(27) $$l_{2}=\sum_{j=1}^{k}V_{2_{j}}$$

(28) $$De_{j}-\overset{¯}{De}\leq V_{2_{j}},\;j=1,...,k$$

(29) $$\overset{¯}{De}-De_{j}\leq V_{2_{j}},\;j=1,...,k$$

(30) $$V_{2_{j}}\;\geq 0,\;j=1,...,k$$

Constraints (5)-(6), and (20)-(23) are also included in this model. The optimal solution of this model indicated as $l_{2}^{⁎}$, is an IDP for $f_{2}$ in the BO model.

#### 4.2.4 $LSI_{2}^{b}$, linearized SO model based on the second approach to find an IDP for $f_{2}$ in the BO model

Using non-negative variables $V_{3_{j}}$ and $V_{4_{j}}$, j=1,...,k, objective function $f_{2}$ is linearized. To be minimized, the objective function of this model is determined as in Eq. (31), taking Constraints (32)-(33) into consideration, as well as Constraints (5)-(6), (20)-(23). The optimal solution of this model denoted as $l_{2}^{⁎}$, is an IDP for $f_{2}$ in the BO model.

(31) $$l_{2}=\sum_{j=1}^{k}V_{3_{j}}+\sum_{j=1}^{k}V_{4_{j}}$$

(32) $$De_{j}-\overset{¯}{De}-V_{3_{j}}+V_{4_{j}}=0,\;j=1,...,k$$

(33) $$V_{3_{j}}\;and\;V_{4_{j}}\geq 0,\;j=1,...,k$$

The number of added variables and constraints in the $LSI_{2}^{a}$ and $LSI_{2}^{b}$ models is the same as described at the end of Subsection 4.2.2.

#### 4.2.5 $LSA_{1}^{a}$, SO model with linearized objective function based on the first approach to find an ADP for $f_{1}$ in the BO model

The objective function of this model is defined as in Eq. (16), to be maximized, considering Constraints (17)-(19), in addition to Constraints (5)-(6). Specified as $l_{1}^{⁎⁎}$, the optimal solution of this model is an ADP for $f_{1}$ in the BO model.

#### 4.2.6 $LSA_{1}^{b}$, SO model with linearized objective function based on the second approach to find an ADP for $f_{1}$ in the BO model

To be maximized, the objective function of this model is determined as in Eq. (24), considering Constraints (25)-(26), in addition to Constraints (5)-(6). Denoted as $l_{1}^{⁎⁎}$, the optimal solution of this model is an ADP for $f_{2}$ in the BO model.

#### 4.2.7 $LSA_{2}^{a}$, SO model with linearized objective function based on the first approach to find an ADP for $f_{2}$ in the BO model

The objective function of this model is as in Eq. (27), to be maximized, regarding Constraints (5)-(6), (28)-(30). Shown as $l_{2}^{⁎⁎}$, the optimal solution of this model is an ADP for $f_{2}$ in the BO model.

#### 4.2.8 $LSA_{2}^{b}$, SO model with linearized objective function based on the second approach to find an ADP for $f_{2}$ in the BO model

To be maximized, the objective function of this model is specified as in Eq. (31), regarding Constraints (32)-(33), in addition to Constraints (5)-(6). Indicated as $l_{2}^{⁎⁎}$, the optimal solution of this model is an ADP for $f_{2}$ in the BO model.

As mentioned before, since Constraints (7)-(8) identify upper limits, are not applicable for maximization. Therefore, the $LSA^{a}$ and $LSA^{b}$ models don't include them.

### 4.3 Redefining the BO model

Since it is not easy to deal with multiple objective functions simultaneously, in this section the BO model is redefined as an SO model using IDPs and ADPs.

The LP-metric method is used to convert an MO problem to an SO one, in which the total distance to the IDPs is minimized, along with maximizing the distance from the ADPs.

In the next subsection, three models are defined based on the LP-metric method.

#### 4.3.1 LP-metric-NL, an NL model using the outputs of the *SI* and *SA* models and LP-metric method to find efficient solutions for the BO model

Using the results obtained from the NL models, to be minimized, the objective function can be defined as in Eq. (34).

(34) $$f={\left[\sum_{m=1}^{2}|\frac{f_{m}-f_{m}^{⁎}}{f_{m}^{⁎⁎}-f_{m}^{⁎}}{|}^{p}\right]}^{\frac{1}{p}}$$

where $f_{1}$ and $f_{2}$ are as in Eqs. (2) and (3) and $f_{m}^{⁎}$, $f_{m}^{⁎⁎}$ are the outputs of the models $SI_{m}$ and $SA_{m}$, m=1,2, respectively. In this model Constraints (5)-(8) are regarded.

#### 4.3.2 LP-metric-$L^{a}$, a L model using the outputs of the $LSI^{a}$ and $LSA^{a}$ models and LP-metric method to find efficient solutions for the BO model

Applying the outcomes achieved from the L models based on the first approach of linearization, to be minimized, the objective function can be determined as in Eq. (35).

(35) $$f={\left[\sum_{m=1}^{2}|\frac{l_{m}-l_{m}^{⁎}}{l_{m}^{⁎⁎}-l_{m}^{⁎}}{|}^{p}\right]}^{\frac{1}{p}}$$

where $l_{1}$ and $l_{2}$ are as in Eqs. (16) and (27), and $l_{m}^{⁎}$, $l_{m}^{⁎⁎}$ are the outputs of the models $LSI_{m}^{a}$ and $LSA_{m}^{a}$, m=1,2, respectively. Constraints (5)-(6), (17)-(23), (28)-(30) are considered in this model.

#### 4.3.3 LP-metric-$L^{b}$, a L model using the outputs of the $LSI^{b}$ and $LSA^{b}$ models and LP-metric method to find efficient solutions for the BO model

Using the results acquired from the L models based on the second approach of linearization, to be minimized, the objective function can be determined as in Eq. (35), in which $l_{1}$ and $l_{2}$ are as in Eqs. (24) and (31), and $l_{m}^{⁎}$, $l_{m}^{⁎⁎}$ are the outputs of the models $LSI_{m}^{b}$ and $LSA_{m}^{b}$, m=1,2, respectively. Constraints (5)-(6), (20)-(23), (25)-(26), (32)-(33) are considered in this model.

For p=1, p=2, and p=∞, the distances are the street-block (or Manhattan), Euclidean, and Chebyshev. A Pareto optimal solution is obtained for each *p*. But the higher *p*, the higher the complexity. In this study, only p=1 is used to simplify.

## 5 Experimental results

We generate four benchmarks as 200×10, 500×31, 1000×76 and 2000×200, which are pointed out as the Number *of* clients × Number *of* sectors. $Q=\lfloor \frac{n}{k}\rfloor$ is the target number of points per sector. In order to have diversity in the benchmarks, *Q* is equal to 20, 16, 13, and 10, respectively. Parameters *τ* ranges inside the interval [0, 1]. Two-dimensional coordinates of clients and center of sectors and likewise clients' demands, are created according to *N*(50;10) and *U*(10;100), which are normal and discrete uniform distributions, respectively [13], [14], [17], [18].

The proposed models are not very complex, but the solution becomes difficult because the models are basically NL and also the number of variables and constraints in the benchmarks can be high, especially in the L models. For example, in the NL models, the number of the binary variables $Z_{ij}$ is n×k. Because of the Constraints (5), (6), (7), (8) respectively, *n* linear, *k* linear, *k* non-linear and *k* non-linear constraints are included in the model. As an example, the total number of variables and constraints for the model $SA_{1}$ are given in Table 6, where *TV* and *NC* stand for the total number of variables and constraints, respectively.

**Table 6:** Number of variables and constraints in model *SA*_1_ to find IDPs for *f*_1_.

Benchmark	*TV*	*NC*
200×10	2000	230
500×31	15500	593
1000×76	76000	1228
2000×200	400000	2600

Subsections 4.2.2 and 4.2.4 specify how the number of variables and constraints varies in the linear models.

A system with an Intel Core i5 processor, 2.4 GHz with 12 GB of RAM is utilized. IBM ILOG CPLEX Optimization Studio 12.9, GAMS 25.1.2, Lingo 19.0, Pymoo 4.2, and Pulp 2.4 are operated. From GAMS solvers BARON is used for the NL models, while CPLEX is used for L ones. Even if BARON is used for L models it is shifted to CPLEX automatically. In the tables of results, CPLEX refers to IBM ILOG CPLEX Optimization Studio, not the solver in GAMS. For the GA and NSGA-II utilized from the Pymoo library, we set the size of the population and offspring equal to 1000. We use a random sampling method and uniform crossover and mutation [16].

We suppose $0\leq \tau_{eqds}\leq 1$, and $0\leq \tau_{eqde}\leq 1$. Using Constraints (7)-(8), the values of $\tau_{eqds}$ and $\tau_{eqde}$ can be calculated as in Statements (36)-(37):

(36) $$0\leq \tau_{eqds}\leq 1-max(\frac{\left|Dc_{j}-\overset{¯}{Dc}\right|}{\overset{¯}{Dc}}),\;j=1,...,k$$

(37) $$0\leq \tau_{eqde}\leq 1-max(\frac{\left|De_{j}-\overset{¯}{De}\right|}{\overset{¯}{De}}),\;j=1,...,k$$

We employ GA to define the values of these parameters. Such that, running the GA for the model SI, starting from 1 and decreasing by 0.05 each time, the highest value that gives a feasible result is selected. We assign the values of these two parameters equally to manage them easily. Thus, the acquired values are as in Table 7. The reason for using GA for this purpose is that it provides results faster.

**Table 7:** Values of the tightness parameters in benchmarks.

Benchmark	*τ*_*eqde*_	*τ*_*eqde*_
200×10	0.75	0.75
500×31	0.5	0.5
1000×76	0.4	0.4
2000×200	0.1	0.1

The results are presented in Table 8, Table 9, Table 10, Table 11, Table 12, Table 13, Table 14. Using the same distributions we generate alternative instances to verify whether the acquired results depend on the employed benchmarks. Obtained outputs for the alternative benchmarks are given in Table 10. But the results in Table 8, Table 9 and 12-14 are for the main benchmarks.

**Table 8:** Results obtained for the model SI.

Benchmark	Model $SI_{1}$									Model $SI_{2}$							
	Lingo			GAMS			GA			Lingo			GAMS			GA	
	$f_{1}^{⁎}$	ST		$f_{1}^{⁎}$	ST		$f_{1}^{⁎}$	ST		$f_{2}^{⁎}$	ST		$f_{2}^{⁎}$	ST		$f_{2}^{⁎}$	ST
200 × 10	NaN	10:00		**0** (Op)	09:00		292 (F)	00:09		NaN	10:00		**7** (F)	10:00		927 (F)	00:07
500 × 31	NaN	10:00		NaN	10:00		**1398** (F)	00:10		NaN	10:00		NaN	10:00		**3265.55** (F)	00:08
1000 × 76	NaN	10:00		NaN	10:00		**3872.63** (F)	00:33		NaN	10:00		NaN	10:00		**11081.1** (F)	00:34
2000 × 200	NaN	10:00		NaN	10:00		**11933.44** (F)	01:29		NaN	10:00		NaN	10:00		**28450.25** (F)	02:17

**Table 9:** Results obtained for the model LSI.

Benchmark	Model $LSI_{1}^{a}$						Model $LSI_{1}^{b}$						Model $LSI_{2}^{a}$						Model $LSI_{2}^{b}$				
	GAMS			CPLEX			GAMS			CPLEX			GAMS			CPLEX			GAMS			CPLEX	
	$l_{1}^{⁎}$	ST		$l_{1}^{⁎}$	ST		$l_{1}^{⁎}$	ST		$l_{1}^{⁎}$	ST		$l_{2}^{⁎}$	ST		$l_{2}^{⁎}$	ST		$l_{2}^{⁎}$	ST		$l_{2}^{⁎}$	ST
200 × 10	**0** (Op)	00:13		**0** (Op)	00:30		**0** (Op)	01:02		**0** (Op)	00:30		**5** (F)	10:00		**5** (F)	10:00		**5** (F)	10:00		**5** (F)	10:00
500 × 31	**20** (F)	10:00		26.06 (F)	10:00		**17** (F)	10:00		61.10 (F)	10:00		71 (F)	10:00		**55.54** (F)	10:00		55 (F)	10:00		**40.45** (F)	10:00
1000 × 76	**222** (F)	10:00		306.63 (F)	10:00		**158** (F)	10:00		593 (F)	10:00		**202** (F)	10:00		268 (F)	10:00		**214** (F)	10:00		366 (F)	10:00
2000 × 200	**857** (F)	10:00		NaN	10:00		**979** (F)	10:00		NaN	10:00		**2669** (F)	10:00		NaN	10:00		**2791** (F)	10:00		NaN	10:00

**Table 10:** Results of the model LSI for the alternative benchmarks.

Benchmark	Model $LSI_{1}^{a}$						Model $LSI_{1}^{b}$						Model $LSI_{2}^{a}$						Model $LSI_{2}^{b}$				
	GAMS			CPLEX			GAMS			CPLEX			GAMS			CPLEX			GAMS			CPLEX	
	$l_{1}^{⁎}$	ST		$l_{1}^{⁎}$	ST		$l_{1}^{⁎}$	ST		$l_{1}^{⁎}$	ST		$l_{2}^{⁎}$	ST		$l_{2}^{⁎}$	ST		$l_{2}^{⁎}$	ST		$l_{2}^{⁎}$	ST
200 × 10	**0** (Op)	05:55		**0** (Op)	01:47		**1** (F)	10:00		2 (F)	10:00		7 (F)	10:00		**6** (F)	10:00		**5** (F)	10:00		**5** (F)	10:00
500 × 31	71 (F)	10:00		**43.87** (F)	10:00		**55** (F)	10:00		172 (F)	10:00		70 (F)	10:00		**54.25** (F)	10:00		32 (F)	10:00		**30.83** (F)	10:00
1000 × 76	**369** (F)	10:00		656 (F)	10:00		**360** (F)	10:00		NaN	10:00		**293** (F)	10:00		835 (F)	10:00		**255** (F)	10:00		895.55 (F)	10:00
2000 × 200	**2333** (F)	10:00		NaN	10:00		**2347** (F)	10:00		NaN	10:00		**2663** (F)	10:00		NaN	10:00		**3393** (F)	10:00		NaN	10:00

**Table 11:** The results of the three-way ANOVA for the outputs of Table 9.

Source	Sum of Square	Degree of Freedom	Mean Square	F-Value	P-Value
Solver	4837880385	1	4837880385	36643.61	0
Size	15484033011.9	3	5161344337.3	39093.63	0
Model	396864	3	132288	1	0.44
Solver:Size	14439241630	3	4813080543.3	36455.77	0
Solver:Model	505963.9	3	168654.6	1.28	0.34
Size:Model	1275973	9	141774.8	1.07	0.46
Error	1188226.8	9	132025.2		
Total	34764522054.6	31

**Table 12:** Acquired results for the model SA by GA.

Benchmark	Model $SA_{1}$			Model $SA_{1}$	
	$f_{1}^{⁎}$	ST		$f_{2}^{⁎}$	ST
200 × 10	482	00:07		406	00:06
500 × 31	2116.32	00:06		7167.54	00:08
1000 × 76	3872.63	00:33		11081.1	00:33
2000 × 200	12071.99	01:34		11933.44	01:29

**Table 13:** Non-dominated solutions obtained for the benchmarks of the model BO by NSGA-II from Pymoo library.

Benchmark	*f*_1_	*f*_2_	*ND*	*ST*
200 × 10	328.4	940	2	00:05
	423.6	927		
500 × 31	1753.74	4020.64	3	00:07
	1468.06	4274.64		
	1883.8	3265.59		
1000 × 76	3872.63	11081.1	1	00:32
2000 × 200	11933.44	29793	2	01:24
	12071.99	29535.25

**Table 14:** Obtained solutions for the benchmarks of the MO model using LP-metric-NL, LP-metric-*L*^*a*^ and LP-metric-*L*^*b*^ methods.

Benchmark	LP-metric-NL					LP-metric-$L^{a}$					LP-metric-$L^{b}$			
	*f*_1_	*f*_2_	*f*	*ST*		*f*_1_	*f*_2_	*f*	*ST*		*f*_1_	*f*_2_	*f*	*ST*
200 × 10	328.4	940	3.01	00:05		26	27	0.11	10:00		**23**	**25**	**0.10**	10:00
500 × 31	1468.04	4247.64	1.28	00:06		**95**	**217**	**0.05**	10:00		**75**	**220**	**0.05**	10:00
1000 × 76	3872.63	11081.1	2	00:32		**286**	**618**	**0.06**	10:00		355	1074	0.13	10:00
2000 × 200	11140.4	28450.25	3.67	02:17		**1442**	**4367**	**0.25**	10:00		1619	5025	0.32	10:00

Although the models are not complex, it is not easy to find their optimal solutions. This is because they are basically NL and when they are linearized the number of variables and constraints grows. Therefore, the tables of results mostly report a feasible solution.

With GA and NSGA-II metaheuristics, only NL models are solved, because the linearization is done to reduce the complexity of the problem, which is not a handicap for these algorithms and they can easily solve the NL models. In the tables, *ST* stands for solution time in a format as minutes:seconds (mm:ss). Optimal results are displayed as *Op*. The maximum working time of the solvers is defined as 10 minutes. If no solution is obtained within this time, it is shown in the tables as NaN. If only a feasible solution is found in 10 minutes, this is signed by *F*.

As seen in Table 8, only GA is able to find solutions for all benchmarks of the model SI within the defined time, though their quality is not satisfactory. Although Pulp and Lingo are capable of solving L models, they only find results for the benchmark with the smallest size. Hence, their outcomes are not presented in Table 9. In the L models, although GAMS uses the CPLEX solver, as seen in Table 9, Table 10, there is a difference between its results and the IBM ILOG CPLEX Optimization Studio. It is observed that GAMS achieves better results for more benchmarks. Moreover, according to the results, linearization based on the first and second approaches does not seem to predominate each other. In Table 8, Table 9, Table 10 the best achieved solution for each model is bold.

To understand if there are significant differences between the results of the solvers, we perform an analysis of variance (ANOVA) with a confidence level of 95%. We use the outputs of Table 9. We define factors as the type of solver, size of the benchmark, and model. So, the analysis is a three-way ANOVA. The null hypothesis is that the results of groups of factors are equal, against the alternative hypothesis that at least one of them is different.

As seen in Table 11, since the p-values for the factors of solver and size are less than 0.05, they are significant. However, the p-value for the factor of the model is greater than 0.05, and consequently, it is not significant. This is also valid for the interactions of the factor of the model with other ones, which are shown as Solver:Model and Size:Model.

While the ANOVA indicates a significant overall difference, it does not determine which specific groups differ from each other. To compare groups, we perform a multiple comparison test, which is a post hoc analysis. The p-value for the test is equal to zero. Two groups are formed based on the results of GAMS and IBM ILOG CPLEX Optimization Studio solvers, whose marginal means of groups are significantly different. Overall, based on the results of the ANOVA and multiple comparison tests, we can conclude that GAMS and IBM ILOG CPLEX Optimization Studio perform differently.

**Figure 3:**
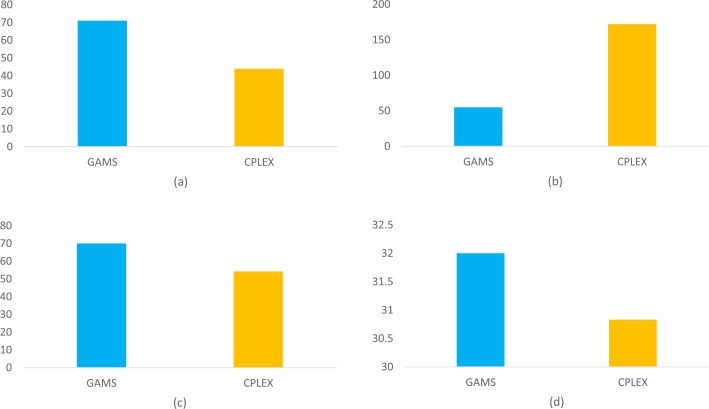
Visualization of the results of benchmark 500 × 31 for Models (a) $LSI_{1}^{a}$, (b) $LSI_{1}^{b}$, (c) $LSI_{2}^{a}$ and (d) $LSI_{2}^{b}$.

As seen in Fig. 3a-d, except for Model $LSI_{1}^{b}$ of benchmark 500 × 31, IBM ILOG CPLEX has better performance than GAMS. But as it appears in Table 10, GAMS outperforms IBM ILOG CPLEX for other benchmarks.

It should be noted that for the LP relaxation of the LSI model, in all benchmarks, both GAMS and IBM ILOG CPLEX Optimization Studio find the optimal result in less than one minute. What is meant by LP relaxation is just that the binary decision variable in Eq. (1) is defined continuously in the interval [0, 1].

GAMS, IBM ILOG CPLEX Optimization Studio, and Lingo could not provide solutions for the SA and LSA models, reporting that the models are unbound. It may be useful to apply additional constraints to avoid this, but this is not the subject of this study. Because the aim of this work is to solve the presented model with existing constraints. Nevertheless, the employed GA gives results for SA as in Table 12, which are supposed as ADPs.

As mentioned before, the NL types of the BO model are also solved with NSGA-II available in the Pymoo library of Python. The results are given in Table 13, where *ND* shows the number of obtained non-dominated solutions.

It has been mentioned earlier that the optimal solutions of the SO models will be utilized as IDPs and ADPs for the BO model, while according to the acquired results, most of them are from the feasible ones. This can be regarded as a limit for the study. Using the IDPs ve ADPs in the LP-metric method, defining p=1, outputs are gained as in Table 14.

**Figure 4:**
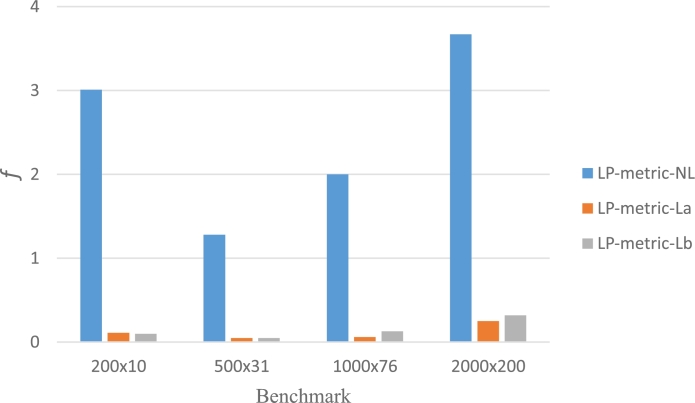
Visualization of the results presented in Table 14.

In addition to $f_{1}$ and $f_{2}$, the values of *f* are also given, which is defined as in Eqs. (34) and (35). As it has been mentioned earlier, the results in Table 12 are regarded as ADPs in all models and $f_{m}^{⁎⁎}=l_{m}^{⁎⁎}$, m=1,2. The LP-metric-NL is solved with GA while the LP-metric-$L^{a}$ and LP-metric-$L^{a}$ are handled with GAMS. Related outputs are presented in Table 14 and Fig. 4, where non-dominated solutions are bold. As seen, some of the non-dominated solutions achieved by the LP-metric method for benchmarks 200×10, 500×31, 1000×76 are the same as those in Table 12. Even for benchmarks 2000×200, the solution provided by the LP-metric-NL method dominates the ones in Table 12. Though, the results of the LP-metric-$L^{a}$ and LP-metric-$L^{b}$ are significantly better than the LP-metric-NL, which is also due to the performance of the CPLEX solver in GAMS.

**Figure 5:**
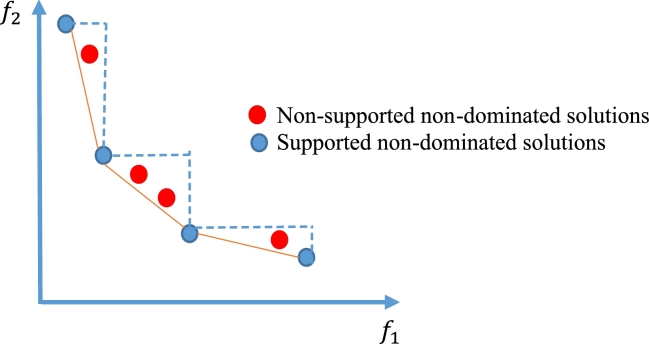
Supported and non-supported non-dominated solutions.

As mentioned earlier, the reason for the definition as p=1 is to don't ascend the complexity of the problem. But consequently, some of the solutions on the Pareto front are overlooked. It is possible to find other non-dominated solutions with different values of *p* [65]. The models designed based on the LP-metric method can only achieve a few of the supported non-dominant solutions. Such solutions are located on the smallest convex polyhedron that includes the feasible region, which is called the convex hull. Whereas the unsupported ones are inside the convex hull. Examples of these solutions are depicted in Fig. 5. Unlike the unsupported non-dominant solutions, the supported one can be uncovered by applying weighted sum or similar methods like LP-metric. Discovering unsupported non-dominant solutions is a challenge for mathematical programming techniques [66], [67], [68]. Based on the similarity between the LP-metric approach and the weighted sum method, it can be emphasized that it is not always possible to identify all non-dominated solutions using this approach.

## 6 Conclusion and future research directions

In this study, for the first time in the literature, a comparison is made between various solvers like Lingo, IBM ILOG CPLEX Optimization Studio, GAMS, and also Pulp, and Pymoo libraries from Python for solving SP. The comparison is made based on a new BO model for an SP. The difficulties of solving the model, which is basically NL, are discussed. The effects of objective functions and the non-linearity of some constraints on this difficulty are mentioned. An approach that had not been used in the literature before is used to solve the model, in which, at first, the BO model is divided into two NL SO models. The results of the SO models, when the objective function is to be minimized and maximized, are assumed to form IDPs and ADPs for the respective objective function in the BO model. Also, the models, which are basically NL, are linearized to solve the BO model easier. Both mathematical programming and metaheuristics are used for the solution. Although the models in the study are not complex, solutions are difficult due to non-linearity and also because of the large number of variables in some benchmarks when they are linearized. For these reasons, solvers are not very efficient in solving models, and in general, during the defined run time, they could not find optimal solutions. Solvers performed better on the L models than the NL ones. Usually, GAMS is known to be one of the best software for operational research. In this study, GAMS provides better results than Lingo and Pulp. Even though GAMS operates the same solver as IBM ILOG CPLEX Optimization Studio for L models, it is slightly better. Among the solvers, the weakest performance is for Pulp.

The applied SO and MO metaheuristics are able to yield feasible solutions in reasonable times though they couldn't find optimal results. Their run times are much less than the other solvers but the quality of the results is not satisfactory. One of the reasons for this matter can be that the used metaheuristics are from a generic open-source library and they are not designed problem-oriented. Nevertheless, it may be useful to use the algorithms of such libraries for solving large-scale problems, where it is not easy to find optimal solutions.

The models defined based on the LP-metric method achieve good results in terms of providing a non-dominated solution for each benchmark. Therefore, it would be said that the approach used for the first time in the literature of MO SP can be useful in finding non-dominant solutions. If more types of distance are used in the formulation, it could be possible to acquire more non-dominated solutions, but in this case, the complexity of the models would increase. This can be seen as a limit for this method. Notwithstanding, it can be concluded that the approach suggested in this study for solving MO SP is practical. The approach can be summarized as follows: dividing the BO model into some SOs ones, finding IDP and ADP for each objective function, reformulating and solving the BO model, and using the IDPs and ADPs in the LP-metric approach.

A limitation of this study is that IDPs and ADPs are got from different approaches. It is reported by the solvers that the maximization of the models is unbound. Although this matter is not an impediment to the completion of this work, it showed that it is better to reformulate the models, in future studies.

Figuring out the deficiencies of the metaheuristics employed in this study, in future studies, it is planned to propose a metaheuristic specific to solve MO SPs, in order to cope with the difficulties arising from a large number of variables and non-linearity in the models.

Performance metrics are used for quantifying performance and comparison of MO methods. It is crucial to use metrics to do comparisons between MO EAs designed based on the concept of Pareto optimality. In most MO problems, the Pareto optimal set is unattainable, correspondingly the measure of performance is challenging. Pareto front generation in MO problems needs high computational effort, especially in large-size problems. So its formation in a short time and with a small memory represents the efficiency of the employed algorithm. Convergence, coverage, and success rate measures are used to compare MO algorithms designed based on the Pareto approach. There are also other measures based on accuracy and variance. Accuracy shows generated non-dominated and the best-known solutions closeness. Coverage and variance have alike definitions. Coverage indicates solutions diversity and distribution. Variance represents the non-dominated front maximum range, which is covered by the generated solutions, for each objective. Diversity-based criteria measure the distances of the solutions in the first front of the final population. This front solution set is compared with a uniform distribution and its deviation is calculated. If an algorithm achieves more uniformly distributed solutions with less average deviation from the first front, it has good performance. Furthermore, criteria are categorized as quality, spacing, and diversification metrics. The obtained non-dominated solutions of algorithms are put together and their ratio is calculated as the quality metric. Spacing metric measures the uniformity of the solutions, while the diversification metric measures the spread of the solution set. There are also metrics such as net front contribution ratio and mean relative percentage increase to measure non-dominated solution set quality [69], [70], [71], [72], [73], [74], [75], [76].

**Figure 6:**
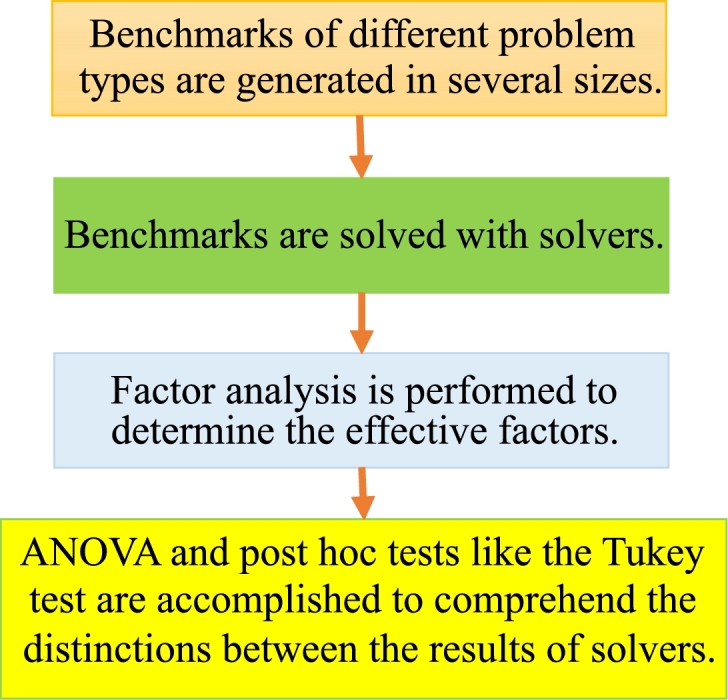
A framework for comparison between the performance of solvers.

A significant limitation of the study is that the comparisons are over limited benchmarks and without a comprehensive statistical design of experiments [13]. In particular, this matter gains importance as the performance variability of the solvers is evident in the results. Likewise, in addition to SPs, benchmarks of similar problems such as p-median [77] should also be contained in the comparison. Statistical analyzes should be performed within a certain confidence interval, taking into account the different levels of various factors which can be problem type, benchmark size, solution, and linearization method [78]. The impacts of factors can be evaluated by factor analysis. The discrepancy between solvers' performance should be explored by designing hypotheses, performing ANOVA, and also post hoc methods such as Tukey's test [79], [80]. A possible framework is summarized in Fig. 6.

Currently, there are not enough studies about the performance of solvers in the literature. In case of existing enough studies on this subject, a meta-analysis can be done, which is a research approach that can explore publications' bias and can draw a broader conclusion. It employs statistical methods to quantitatively compare and also combines the results of multiple studies [81], [82].

In the literature, not enough attention has been given to the MADM techniques outlined in Fig. 2 to solve SPs. In real-life problems, DMs usually assign dissimilar priorities to criteria, in which case both compensatory and non-compensatory MADM can be beneficial.

Integrating MIP techniques with neural networks can provide an intelligence search on the feasible space [83], [84], which will be considered in future works.

## CRediT authorship contribution statement

Aydin Teymourifar: Conceived and designed the experiments; Performed the experiments; Analyzed and interpreted the data; Contributed reagents, materials, analysis tools or data; Wrote the paper.

## Declaration of Competing Interest

The authors declare that they have no known competing financial interests or personal relationships that could have appeared to influence the work reported in this paper.

## Figures and Tables

**Table:** 

Abbreviations	Definition
ADP	Anti-ideal point for an objective function
AHP	Analytical hierarchy process
ANOVA	Analysis of variance
ANP	Analytic network process
BO	Bi-objective
DM	Decision maker
EA	Evolutionary algorithm
F	Feasible solution
GA	Genetic algorithm
GP	Goal programming
IDP	Ideal point for an objective function
ILP	Integer linear programming
Inf	Infimum
INLP	Integer non-linear programming
IQP	Integer quadratic programming
L	Linear
LP	Linear programming
LSA	Single-objective model with linearized objective function to find anti-ideal points of the bi-objective model
LSI	Single-objective model with linearized objective function to find ideal points of the bi-objective model
MADM	Multi-attribute decision making
MCDM	Multi-criteria decision making
MILP	Mixed integer linear programming
MINLP	Mixed integer non-linear programming
MIP	Mixed integer programming
MO	Multi-objective
MODM	Multi-objective decision making
NaN	No solution obtained
NC	Number of constraints
ND	Number of obtained non-dominated solutions
NL	Non-linear
NLP	Non-linear programming
NSGA-II	Non-dominated sorting genetic algorithm
Op	Optimal solution
p	Emphasis level in the LP-metric method
Pa	Pareto solution
SA	Single-objective model to find anti-ideal points of the bi-objective model
SI	Single-objective model to find ideal points of the bi-objective model
SO	Single-objective
SP	Sectorization problem
ST	Solution time
STEM	Step method
Sup	Supremum
SWTM	Surrogate worth trade-off method
TOPSIS	Technique for Order Preference by Similarity to Ideal Solution
TV	Total number of variables

## Data Availability

Data will be made available on request.

## Appendix A

Table of abbreviations
